# Assessment of the impact of nonavalent human papillomavirus vaccine on disease clearance time in women with active infection

**DOI:** 10.61622/rbgo/2026rbgo17

**Published:** 2026-05-29

**Authors:** Nina Grillo, Rita Almendra, Cátia Correia

**Affiliations:** 1 Unidade Local de Saúde do Alto Alentejo Hospital Santa Luzia de Elvas Elvas Portugal Hospital Santa Luzia de Elvas, Unidade Local de Saúde do Alto Alentejo, Elvas, Portugal.; 2 Unidade Local de Saúde de Braga Hospital de Braga Braga Portugal Hospital de Braga, Unidade Local de Saúde de Braga, Braga, Portugal.

**Keywords:** Human papillomavirus viruses, Papillomavirus infections, Papillomavirus vaccines, Squamous intraepithelial lesions, Uterine cervical neoplasms

## Abstract

**Objective:**

Human papillomavirus (HPV) is a leading cause of cervical cancer (CC) worldwide. There is growing interest in the potential role of HPV vaccination as adjuvant measure in patients with active infection. This study aimed to assess whether the nonavalent HPV vaccine (9vHPV) promotes faster disease clearance in women with low-grade (LGD) and high-grade disease (HGD).

**Methods:**

Retrospective study including women aged 25–60 years who began follow-up in 2018 at a central Portuguese hospital after referral through organized CC screening. A total of 126 patients with HGD (69 vaccinated, 57 unvaccinated) and 277 with LGD (131 vaccinated, 146 unvaccinated) at baseline were followed with HPV cotesting (HPV test + cervical cytology) at 12, 18, 24, 36, and 48 months. Disease clearance was defined as the first negative cotest result. Kaplan-Meier curves and the Log Rank test were used to compare time to clearance between groups.

**Results:**

Among HGD patients, the vaccinated group showed a shorter mean time to disease clearance (17.4 months; 95% CI: 14.88–19.93) compared to the unvaccinated group (20.2 months; 95% CI: 17.07–23.34), although the difference was not statistically significant (p=0.228). In the LGD group, both vaccinated and unvaccinated patients had similar mean clearance times (25.9 months; 95% CI: 23.44–28.43 vs. 26.9 months; 95% CI: 24.41–29.37), also lacking statistical significance (p=0.637).

**Conclusion:**

Main findings suggest a negligible effect of 9vHPV in significantly reducing time to clearance in patients with active infection. Further research is needed to clarify its role in secondary prevention strategies.

## Introduction

Human papillomavirus (HPV) is the most common agent of sexually transmitted disease, being responsible for the great majority of cervical cancer (CC) cases on a global scale. There are more than 200 different genotypes of HPV identified to date, which can be classified by their potential to promote malignant transformation. There are 12 HPV types defined as oncogenic, also known as high-risk (HR) HPVs, namely HPVs 16, 18, 31, 33, 35, 39, 45, 51, 52, 56, 58, and 59, and several types as probably oncogenic including types 66 and 68.^(1)^ HPVs 16 and 18 collectively account for 71% of all cases of CC worldwide, being considered the most oncogenic types.^(2)^

Even though most HPV infections are asymptomatic and resolve spontaneously within a period of 1-2 years, 5-10% of infected women will develop persistent infection. Persistent HPV infection is a necessary, yet non-sufficient cause for the development of CC, and HR HPV types are more likely to lead to precancerous lesions that may ultimately progress to cancer if the infection is left untreated.^(3)^ Besides HR HPV persistence, there are other predisposing conditions that can contribute to disease progression, including tobacco exposure, compromised immune status, concurrent infection with other sexually transmitted infections, multiple parity, and use of oral contraceptives.^(4)^

Since CC has a long evolution time and is preceded by treatable precancerous lesions, prevention initiatives have been globally implemented with the goal of decreasing morbidity and mortality associated with the disease. CC can be prevented through implementation of primary (HPV vaccination), secondary (screening and treatment of precancerous lesions), and tertiary (early cancer diagnosis and treatment) measures, that should be combined to reduce disease burden.^(5)^

Considering that Top of FormBottom of Formprevious HPV infection does not usually confer a sufficient immune response to protect against future infections, even in case of reactivation or reinfection with the same HPV genotypeTop of FormBottom of Form,^(6)^ latest evidence strongly favours universal HPV vaccination, as the most effective and cost-efficient strategy to accelerate the elimination of HPV-related diseases, including CC.^(7)^ Currently in Portugal it is recommended that boys and girls are vaccinated with two doses of nonavalent (9vHPV) HPV vaccine — targeting HPVs 16/18/31/33/45/52/58/6/11, to be administered at 10 years of age, while catch-up vaccination beyond 15 years is usually done with three 9vHPV doses.^(8)^ Top of FormBottom of Form

Even though HPV vaccines act best if administered before sexual debut,^(9)^ providing prophylactic action against covered HPV types, their licensing, medical indications, and dosing schedules continue to be updated.^(10)^ The United States Food and Drug Administration has extended the upper age limit of 9vHPV to 45 years in both women and men since 2018.^(11)^ The European Union vaccine licencing does not have an upper age limit for HPV vaccination.^(12-14)^ Although no formal recommendation has yet been issued to expand catch-up HPV vaccination beyond 26 years of age, the Advisory Committee on Immunization Practices of the United States recommends patient-clinician shared decision-making regarding vaccination between 27-45 years since 2019.^(15)^

Given the heavy disease burden associated with HPV infection, the scientific community has recently shown particular interest about the potential role of HPV vaccination in reducing the risk of disease recurrence in patients with active HPV infection. Although published literature on this subject is limited and the efficacy of HPV vaccination as adjuvant measure remains controversial, with no HPV vaccine yet approved for therapeutic use, the increasing new evidence within this scope could lead to new perspectives in the management of HPV-related diseases.^(16-19)^

## Methods

This project was designed to investigate the impact of 9vHPV in women with HPV-related low and high-grade disease, comparing disease clearance time, disease persistence, and progression to higher grade lesions during follow-up between vaccinated and unvaccinated women. It involved all the women who started follow-up at a central hospital in northern Portugal in 2018, after being referred by their primary care practitioner because of abnormal test results obtained in the framework of the Portuguese organized CC screening (PT-CCS).

Inclusion criteria were applied as follows:

- Satisfaction of eligibility and referral criteria of the PT-CCS.
- Positive for one or more HPV types diagnosed in the framework of the PT-CCS.
- Date of first cervical pathology consultation within the year of 2018.
- Colposcopy and/or cervical biopsy were performed at baseline.
- Patients with confirmed high-grade disease at baseline were treated with excision of the transformation zone (ETZ).
- Patients with suspected low-grade disease at baseline were not submitted to ETZ.
- Minimum follow-up time of 12 months.
- Regarding vaccinees: completion of 9vHPV 3-dose schedule up until 12 months of follow-up.

Exclusion criteria were the following:

- Histologically confirmed invasive CC at baseline.
- Patient underwent total hysterectomy at baseline or during follow-up.
- Immunocompromised status.
- Unknown vaccination status.

**Figure 1:**
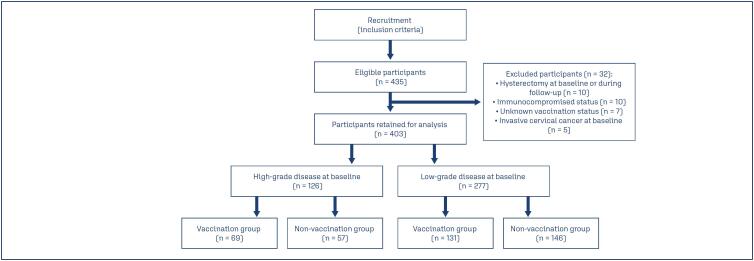
Inclusion and distribution of participants in the present study

The records of all 435 patients, aged 25–60 years, who fulfilled the inclusion criteria were retrospectively reviewed. A total of 403 patients were considered eligible for the study (126 high-grade disease, 277 low-grade disease patients), while 32 patients were removed from the analysis after application of the exclusion criteria (Figure 1).

Eligible patients were divided into two different study populations according to baseline grade of disease. Lower anogenital squamous terminology (LAST) nomenclature was used for colposcopic biopsy diagnoses.^(20)^ Women with histologically confirmed HSIL at baseline were considered as having high-grade disease, while women with negative colposcopy and/or histological LSIL result or lower were classified as low-grade. Since high-grade disease is treated surgically, the date of the first ETZ was considered as baseline for high-grade patients. For low-grade patients, baseline was set as the date of initial colposcopy. Given the differences in treatment and follow-up approach according to disease grade, low and high-grade disease patients were considered two different study populations for statistical analysis and were never compared with each other. Each study population was then further divided into two study groups, according to HPV vaccination status.

Vaccinees were defined as women vaccinated with 3 doses of 9vHPV up until 1 year after baseline (vaccination group). Women who were followed without any vaccination were described as non-vaccinees (non-vaccination group). Vaccination costs represented an out-of-pocket expense for patients, not being covered by the hospital.

Follow-up visits were carried out at 12, 18 and 24 months after baseline for the first 2 years, and yearly thereafter for up to a total of 4 years (12, 18, 24, 36 and 48 months). HPV cotesting (HPV test + cervical cytology) was the preferred testing method for cervical surveillance.^(21)^

HPV cotest results were categorized as follows:

- HPV test: Cobas® 4800 HPV Test System was used to detect a total of 14 HR HPV types: HPV 16 and 18 individually, and 12 additional HR HPV genotypes (31, 33, 35, 39, 45, 51, 52, 56, 58, 59, 66, and 68) pooled as "other". HPV cotests with any detected HR HPV types during follow-up were described as HPV positive.
- Cervical cytology: Bethesda classification was used for cytological diagnoses.^(22)^ ASC-H, AGC and HSIL results were grouped as high-grade lesions, ASC-US and LSIL were grouped as low-grade lesions, and NILM results were described as normal.

Disease clearance was defined as the first negative cotest result (HPV negative + NILM) obtained during follow-up. For time-to-event analysis, disease clearance was considered the event of interest, being treated as the primary endpoint. For all participant who achieved clearance, time elapsed between baseline and disease clearance was recorded in months, based on follow-up timings. All other data were censored at the last recorded visit, occurrence of an event that would preclude or impact further follow-up assessments (loss to follow-up, referral to ETZ treatment), or at 48 months, whichever occurred first.

Criteria for disease persistence and progression were based on cotest and histological results. Patients with positive cotest results for low-grade lesions and/or HPV positivity for a minimum of 2 years, that is at least at 12 and 24 months consecutively, were considered as having persistent disease. Any positive cotest result for high-grade lesions obtained during follow-up and confirmed histologically by cervical biopsy or ETZ was considered as disease progression or recurrence in patients with previous low-grade and high-grade disease, respectively.

Statistical analysis was performed using IBM's Statistical Package for the Social Sciences®. Differences between groups (vaccination vs non-vaccination) regarding continuous and discrete variables were analysed by Student's t-test or Mann-Whitney U test as appropriate. Categorical variables were compared using Fisher's Exact test. Kaplan-Meier statistical analysis was used to estimate overall time to clearance during follow-up period. Time-to-event curves of both study groups were compared with the Log Rank test. Univariate analyses were carried out to evaluate potential factors associated with disease clearance, persistence and progression/recurrence. Cox proportional-hazards model was used to conduct a final multivariable analysis using a subset of baseline variables to control for potential confounding effects stemming from the patients’ characteristics. Hazard ratios (HR) and their corresponding 95% confidence intervals (CIs) were calculated. Additionally, incidence of disease persistence and progression/recurrence during follow-up was compared between both study groups using Pearson Chi-Square test. All reported p values are two-sided and were considered statistically significant if <0.05.

All relevant information was collected by accessing clinical records through Glintt Healthcare Solutions® medical software. Ethical approval for this study was obtained from the hospital's Data Protection Officer and Health Ethics Committee and from the Ethics Committee for Research in Life and Health Sciences of the University of Minho (CEICVS 160/2023).

## Results

A summary of the characteristics from the 403 participants, for which baseline data was available, can be seen in table 1 according to vaccination status. Distribution of HR HPV genotypes at baseline can be seen in table 2.

**Table 1:** Patient characteristics

Characteristics		High-grade disease patients (n=126)			Low-grade disease patients (n=277)		
		Vaccination group (n=69)	Non-vaccination group (n=57)	p-value	Vaccination group (n=131)	Non-vaccination group (n=146)	p-value
Age (years)				0.111			<0.001
	Median (P25-75)	36.00(32.00-44.00)	40.00(35.00-45.50)		40.00(32.00-45.00)	44.00(36.00-50.00)	
	Range	26-60	26-60		25-58	26-60	
Age at fist intercourse (years)				0.750			0.713
	Median (P25-75)	18.00(17.00-20.00)	19.00(17.00-20.00)		19.00(17.00-21.00)	19.00(17.75-20.25)	
	Range	15-28	14-25		10-33	14-51	
Number of lifetime sexual partners				0.671			0.845
	Median (P25-75)	3.00(1.00-5.00)	2.00(1.00-4.00)		2.00(1.00-4.00)	2.00(1.00-5.00)	
	Range	1-30	1-7		1-12	1-20	
Parity				0.366			0.002
	Median (P25-75)	1.00 (0.00-2.00)	1.00 (0.00-2.00)		1.00 (0.00-2.00)	1.00 (0.00-2.00)	
	Range	0-3	0-5		0-3	0-6	
Tobacco exposure				0.236			0.843
	Current smoker n(%)	16(23.2)	15(26.3)		30(22.9)	34(23.3)	
	Former smoker n(%)	0(0)	1(1.7)		3(2.3)	4(2.7)	
	Never smoked n(%)	49(71.0)	29(50.9)		89(67.9)	88(60.3)	
	Missing data n(%)	4(5.8)	12(21.1)		9(6.9)	20(13.7)	
Type of HPV infection				0.565			0.123
	Single n(%)	41(59.4)	37(64.9)		80(61.1)	100(68.5)	
	Multiple n(%)	28(40.6)	20(35.1)		51(38.9)	46(31.5)	
Baseline HPV16 or 18				0.263			0.942
	Positive n(%)	49(71.0)	37(64.9)		84(64.1)	93(63.7)	
	Negative n(%)	20(29.0)	20(35.1)		47(35.9)	53(36.3)	
Baseline 9vHPV HR HPVs				0.780			0.997
	Positive n(%)	62(89.9)	50(87.7)		105(80.2)	117(80.1)	
	Negative n(%)	7(10.1)	7(12.3)		26(19.8)	29(19.9)	

HPV - human papillomavirus; P25-75 - percentiles; 9vHPV - nonavalent HPV vaccine; HR - high-risk. The variables "age" and "age at fist intercourse" were evaluated for normality through the Kolmogorov-Smirnov test, and by visual inspection of Q-Q plots and histograms, and none followed a normal distribution. Therefore, differences between groups (vaccination vs non-vaccination) regarding continuous and discrete variables were analysed by Mann-Whitney U test. Categorical variables were compared using Fisher's Exact test.

**Table 2:** HR HPV genotypes present at baseline infection

HR HPV genotypes present at baseline infection		High-grade disease patients			Low-grade disease patients		
		Vaccination group (n=69)	Non-vaccination group (n=57)	Total (n=126)	Vaccination group (n=131)	Non-vaccination group (n =146)	Total (n=277)
HR HPVs targeted by 9vHPV (n=334)							
	16	33/62	21/50	54/112	46/105	65/117	111/222
	18	4/62	3/50	7/112	13/105	8/117	21/222
	31	4/62	5/50	9/112	6/105	7/117	13/222
	33	3/62	2/50	5/112	2/105	3/117	5/222
	45	0/62	1/50	1/112	0/105	2/117	2/222
	52	2/62	3/50	5/112	7/105	7/117	14/222
	58	2/62	1/50	3/112	5/105	3/117	8/222
	Multiple infection including >1 9vHPV HR HPV	14/62	14/50	28/112	26/105	22/117	48/222
HR HPVs not targeted by 9vHPV (n=69)							
	35	1/7	0/7	1/14	1/26	0/29	1/55
	39	0/7	1/7	1/14	3/26	3/29	6/55
	51	1/7	2/7	3/14	3/26	3/29	6/55
	56	0/7	0/7	0/14	5/26	3/29	8/55
	59	0/7	1/7	1/14	4/26	4/29	8/55
	66	2/7	1/7	3/14	2/26	5/29	7/55
	68	0/7	2/7	2/14	2/26	6/29	8/55
	Multiple infection excluding 9vHPV HR HPVs	3/7	0/7	3/14	6/26	5/29	11/55

HR - high-risk; HPV - human papillomavirus; 9vHPV - nonavalent HPV vaccine

Among high-grade disease patients, median age was 39 years, ranging from 26-60 years. Median age at first intercourse was 18 years, with a median of 3 sexual partners and 1 birth over their lifetime. Additionally, 24.6% of women reported active smoking habits. Concerning baseline HPV infection, in most women (61.9%) there was a single HPV type present in the initial HPV genotyping. In women with multiple HPV infection, 25.4% tested positive for 2 HR HPV types, 8.7% for 3 HR HPV types and 4% for ≥ 4 HR HPV types simultaneously. Considering the HR HPV types involved, most women (66.7%) tested positive at baseline for HPVs 16 or 18.

Of the 126 high-grade disease patients included in the present study, 69 patients received 9vHPV and 57 patients did not receive any vaccination. Baseline demographic characteristics were very similar between patients in the vaccination and non-vaccination groups regarding age, age at first intercourse, number of lifetime sexual partners, parity and tobacco exposure. Type of infection, as well as baseline HPV positivity to HPV 16 or 18 and 9vHPV HR HPVs were also not significantly different between vaccinees and non-vaccinees (p=0.565; p=0.263; p=0.780 respectively) (Table 1).

In low-grade disease patients, median age was slightly higher at 41 years and ranged from 25-60 years. Median age at first intercourse was 19 years and median number of lifetime sexual partners and births were identical to those reported in high-grade disease patients. 23.1% of women were active smokers. 65.0% of women were infected by a single HPV type, while 21.7% tested positive for 2 HR HPV types, 9.7% for 3 HR HPV types and 3.6% for ≥ 4 HR HPV types simultaneously. HPVs 16 or 18 were present in 63.9% of infections.

A total of 277 low-grade disease patients were retained for analysis, among which 131 patients received 9vHPV and 146 patients were followed without vaccination. Both groups were homogenous regarding age at first intercourse, number of lifetime sexual partners, tobacco exposure and baseline infection determinants. However, it is important to note that demographic characteristics between vaccinees and non-vaccinees differed significantly regarding age (p<0.001) and parity (p=0.002) (Table 1). Statistical analysis was adjusted to these variables in the final multivariate model, to mitigate a possible influence of this discrepancy between groups in our results.

Considering HR HPV genotypes present at baseline (Table 2), 88.9% (112/126) of total high-grade disease cases, and 80.1% (222/277) of low-grade patients were related to HR HPV types covered by 9vHPV, while 11.1% (14/126), and 19.9% (55/277), respectively, were caused solely by other HR HPV types not targeted by the vaccine.

**Figure 2:**
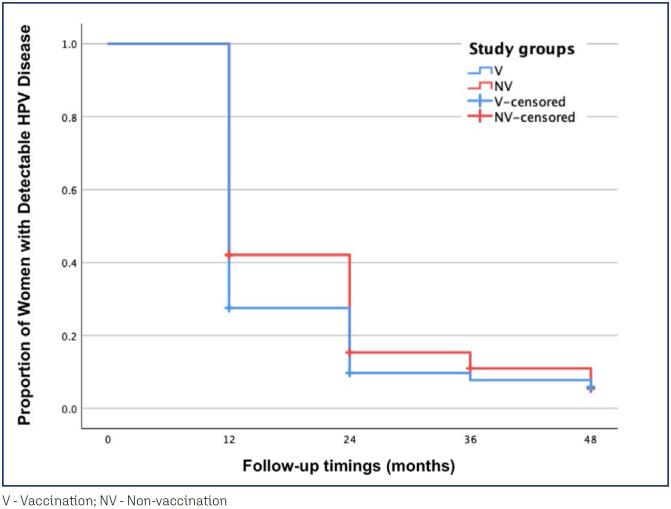
High-grade disease patients: Kaplan-Meier curves for overall time to clearance between vaccinees and non-vaccinees during follow-up

Among high-grade women, overall mean time between baseline and disease clearance was 17.4 months (95%CI:14.88–19.93) in the vaccination group and 20.2 months (95%CI:17.07–23.34) in the non-vaccination group. Although mean clearance time in the vaccination group was lower, Kaplan-Meier curves (Figure 2) showed a statistically non-significant difference in the log-rank test (p=0.228) for the overall time to clearance between both study groups during follow-up.

**Figure 3:**
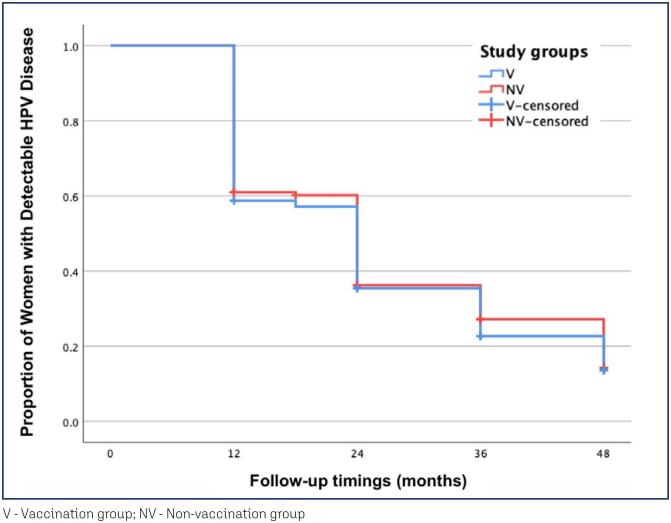
Low-grade disease patients: Kaplan-Meier curves for overall time to clearance between vaccinees and non-vaccinees during follow-up

Among low-grade disease women, overall mean clearance time was very similar between both study groups, namely 25.9 months (95%CI:23.44–28.43) in the vaccination group and 26.9 months (95%CI:24.41–29.37) in the non-vaccination group, with no statistical difference in the log-rank test between both Kaplan Meier curves (p=0.637) (Figure 3).

A cumulative distribution of disease clearance achievement in vaccinated and non-vaccinated women during follow-up can be seen in table 3. 90.5% (114/126) of high-grade disease patients and 76.9% (213/277) of low-grade disease patients reached disease clearance during follow-up. Among high-grade disease patients, most women in both study groups obtained their first negative cotest result at 12 months of follow-up, namely 72.5% (50/69) of the vaccination group and 57.9% (33/57) of the non-vaccination group. However, this difference between both groups, analyzed with Chi-square test, did not show a statistical significance (p=0.086). In low-grade disease patients, at 12 months only 41.2% (54/131) of vaccinees and 39.0% (57/131) of non-vaccinees had a negative cotest result. Most women reached disease clearance until 24 months of follow-up in both study groups, and at 48 months 19.8% (26/131) of vaccinees and 26% (38/146) of non-vaccinees still had not obtained a negative cotest result during follow-up, but still with no statistical difference between groups (p=0.254).

**Table 3:** Cumulative distribution of disease clearance during follow-up timings

		Follow-up timings (months)				
		12	18	24	36	48
High-grade disease patients (n=126)						
	Vaccination group (n=69)	50/63	50/63	61/63	62/63	63/63
	Non-vaccination group (n=57)	33/51	33/51	47/51	49/51	51/51
	p-value	0.086	0.086	0.342	0.502	0.728
Low-grade disease patients (n=277)						
	Vaccination group (n=131)	54/105	56/105	83/105	97/105	105/105
	Non-vaccination group (n=146)	57/108	58/108	89/108	98/108	108/108
	p-value	0.715	0.627	0.711	0.236	0.254

Comparison between groups was carried out using Pearson Chi-Square test statistic.

Considering the high-grade disease population, among the 43 women who did not reach disease clearance within 12 months (n=19 in the vaccination group and n=24 in the non-vaccination group), 11 women (n=6 in the vaccination group and n=5 in the non-vaccination group) had low-grade disease persistence, out of which 1 non-vaccinated woman developed high-grade disease recurrence at 36 months and was referred to new surgical treatment. Considering the low-grade disease population, among the 166 women who did not reach disease clearance within 12 months (n=77 in the vaccination group and n=89 in the non-vaccination group), 88 women (n=40 in the vaccination group and n=48 in the non-vaccination group) had low-grade disease persistence, out of which 7 vaccinated and 9 non-vaccinated women developed disease progression, with histologically confirmed high-grade lesions. In both low and high-grade disease populations, persistence, and progression rates among vaccinees and non-vaccinees were homogenous and reported not statically different by Chi-Square test (Table 4).

**Table 4:** Incidence of disease persistence and progression during follow-up period in relation to vaccination status

		Persistence	No persistence	p-value
High-grade disease patients (n=126)				0.988
	Vaccination group (n=69)	6/11	63/115	
	Non-vaccination group (n=57)	5/11	52/115	
Low-grade disease patients (n=277)				0.676
	Vaccination group (n=131)	40/88	91/189	
	Non-vaccination group (n=146)	48/88	98/189	
		**Progression/recurrence**	**No progression/recurrence**	**p-value**
High-grade disease patients (n=126)				0.269
	Vaccination group (n=69)	0/1	69/125	
	Non-vaccination group (n=57)	1/1	56/125	
Low-grade disease patients (n=277)				0.770
	Vaccination group (n=131)	7/16	124/261	
	Non-vaccination group (n=146)	9/16	137/261	

Comparison between groups was carried out using Pearson Chi-Square test statistic

**Figure 4:**
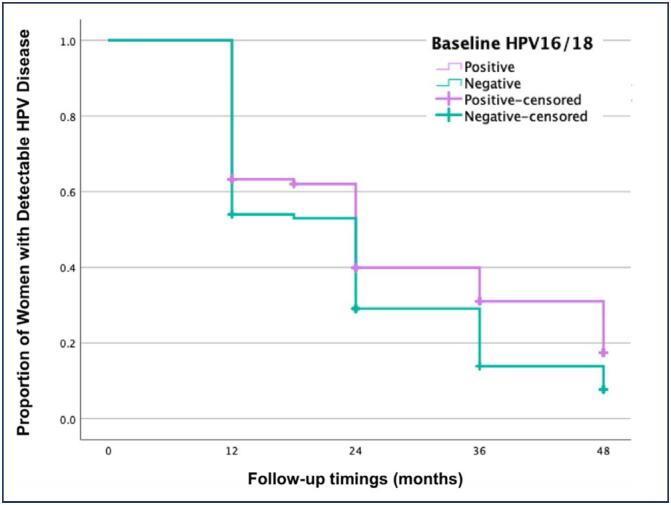
Low-grade disease patients: Kaplan-Meier curves for overall time to clearance during follow-up between women that tested positive and negative for HPV16/18 at baseline

In the low-grade disease population, univariate analysis revealed that irrespective of vaccination status, baseline HPV16/18 positivity was the single risk factor that was significantly linked to a prolonged disease clearance time in the Log Rank test (p=0.011). Mean clearance time was 28.0 months (95%CI:25.74–30.32) among HPV16/18-positive women versus 23.6 months (95%CI:20.97–26.17) in HPV16/18-negative women (Figure 4). HPV16/18 positivity was also identified as an independent risk factor for disease progression (p=0.010). A higher disease persistence rate was also found among HPV16/18-positive women, however this last result did not reach statistical significance (p=0.069) (Table 5). In the high-grade disease population, no risk factors were identified as statistically significant in relation to disease clearance time, disease persistence and recurrence during follow-up.

Finally, a multivariate analysis of disease clearance time among vaccinees and non-vaccinees was conducted with the Cox Regression in the low-grade disease population, to control for potential confounding effects in the initial Kaplan Meier curves stemming from the difference between study groups regarding age and parity, as well as the influence of HPV16/18 positivity in disease clearance times. The final model results, adjusted to the variables age, parity and HPV16/18 positivity can also be seen in table 5. The final model showed that vaccination with 9vHPV was not effective in shortening disease clearance times in low-grade disease patients, even after controlling for other factors (p=0.900). HPV16/18 positivity, however, still had a significant impact on disease clearance times (p=0.048).

**Table 5:** Univariate and multivariate analysis in low-grade disease population

Univariate analysis for potential risk factors influencing disease persistence and progression in low-grade disease population								
			Persistence (n=88)	No persistence (n=189)	p-value	Progression (n=16)	No progression (n=261)	p-value
Low-grade disease patients (n=277)								
	Age (years)				0.187			0.157
		Median (P25-75)	41.00(35.00-50.00)	41.00(33.50-46.50)		43.50(38.50-50.00)	41.00(33.00-47.00)	
		Range	25-60	26-60		31-60	25-60	
	Parity				0.062			0.054
		Median (P25-75)	1.00(1.00-2.00)	1.00(0.00-2.00)		2.00(1.00-2.00)	1.00(0.00-2.00)	
		Range	0-6	0-4		0-4	0-6	
	Tobacco exposure				0.699			0.491
		Current smoker n(%)	20(22.7)	44(23.3)		5(31.3)	59(22.6)	
		Former smoker n(%)	3(3.4)	4(2.1)		0(0)	7(2.7)	
		Never smoker n(%)	52(59.1)	125(66.1)		8(50.0)	169(64.8)	
		Missing data n(%)	13(14.8)	16(8.5)		3(18.7)	26(9.9)	
	Baseline HPV16 or 18				0.069			0.010
		Positive n(%)	63(71.6)	114(60.3)		15(93.8)	162(62.1)	
		Negative n(%)	25(28.4)	75(39.7)		1(6.3)	99(37.9)	
	9vHPV				0.676			0.770
		Vaccinees n(%)	40(45.5)	91(48.1)		7(43.7)	124(47.5)	
		Non-vaccinees n(%)	48(54.5)	98(51.9)		9(56.3)	137(52.5)	
**Multivariable Cox Regression of disease clearance time among vaccinees and non-vaccinees during follow-up in low-grade disease population.**								
			**Hazards ratio (95% CI)**		**p-value**			
Age			0.994(0.975-1.014)		0.581			
Parity			0.917(0.783-1.074)		0.282			
Baseline 16 or 18								
	Positive vs Negative		0.757(0.575-0.997)		0.048			
9vHPV								
	Vaccinees vs Non-vaccinees		0.744(0.744-1.297)		0.900			

## Discussion

The importance of HPV vaccination among women with pre-existing infection is highlighted throughout currently available evidence, but research efforts within this scope are still in the process of gathering sufficient and robust data, especially in relation to 9vHPV.

Vaccinating previously infected women is considered a safe practice, that evokes a strong immune response with various benefits, including protection against HPV strains beyond those initially contracted.^(7,15)^ In a recent publication by Vorsters et al.,^(23)^ the authors argue that even in women with active infection, vaccination leads to the production of a significant level of anti-HPV antibodies in cervicovaginal secretions, that are potentially capable of neutralizing the virus, decreasing the likelihood of disease progression, reinfection, as well as sexual transmission.

There are several other recently conducted studies that report favourable outcomes particularly for women with high-grade disease who received HPV vaccination following surgical treatment,^(24,25)^ with significant reduction of disease relapse rates and underlining the role of HPV vaccination in protecting against recurrent high-grade disease.^(16-19,26,27)^

However, emerging evidence around this topic is still inconsistent. A comprehensive prospective registry-based study conducted by Sand et al., in 2020,^(28)^ that included 17.128 women with high-grade lesions, found that although women who received 4vHPV as an adjunct to conization demonstrated a reduced risk of subsequent CIN2+ development when compared to non-vaccinated women, the difference was reported as statistically nonsignificant.

The main findings of the present study suggest a negligible positive effect of 9vHPV in promoting a shorter disease clearance time during follow-up of patients with baseline low or high-grade disease. Regarding disease persistence and progression rates, in both low and high-grade disease populations, absolute persistence and progression numbers among vaccinees and non-vaccinees during follow-up period were homogenous and reported not statistically different, failing to identify 9vHPV vaccination as an independent protective factor against disease persistence and progression. It is to note that among high-grade disease patients, the only reported recurrence case was in a non-vaccinee. However, derived from the small numbers and lack of data variability, this finding should be interpreted with caution. Additionally, in low grade-disease patients, irrespective of vaccination status, baseline HPV16/18 positivity was the single risk factor that was significantly linked to a prolonged disease clearance time and higher progression rate, which is consistent with literature.^(29-31)^

It is clinically relevant to determine to which extent women that have already been exposed to HPV infection can further benefit from HPV vaccination. Considering that women who persist as chronic HR HPV carriers at higher risk for development of CC^(4)^ and demand hospital follow-up, a positive effect of HPV vaccination among previously infected patients could lead to a swifter and safer discharge and return of affected women to primary-care-unit-led follow-up, resulting in reduction of physical and psychological disease burden, as well as healthcare costs associated with diagnostic and therapeutic procedures inherent to infection surveillance.

Prospective research is needed to gain a better understanding of 9vHPV's potential contribution in this setting. To our knowledge there are currently three ongoing randomized controlled trials exploring this topic and utilizing 9vHPV.^(32-34)^ These trials, that all started in 2019, have us awaiting more consolidated data and will hopefully provide more clarity on 9vHPV's secondary prevention role regarding HPV-related disease.

Given the retrospective nature of this study, there are limitations that should be acknowledged. Firstly, while there are well-defined follow-up times for low and high-grade cases according to the current consensus,^(21)^ the total duration of hospital follow-up and discharge timings after a negative cotest result may vary among different physicians. This is one of the reasons why the first negative cotest result had to be considered the primary endpoint in time-to-event analysis, as it was the only universally applicable criterion. Regarding incidence of disease progression/recurrence, the mentioned limitation did also not allow us to analyse disease-free time during follow-up, which is usually the preferred design in this type of analysis. Secondly, since only partial HPV genotyping is performed during control visits, it was not possible to determine if the infection present in each of the follow-up timings was caused exactly by the same HPV genotypes present at baseline. Lastly, even though no strong relationship was found between vaccination and improvement of disease clearance times, as well as disease persistence and progression rates during follow-up, it would be reasonable to consider that the lack of significance of the obtained results might have been influenced by the small sample size and other mentioned constraints.

Because this study retrospectively analyzed the complete cohort of women who initiated cervical pathology follow-up in 2018 at a central Portuguese hospital, the sample size was fixed and no a priori sample-size calculation was performed. To support interpretation of the outcomes, a post-hoc power sensitivity analysis was conducted using the observed number of disease-clearance events and the proportion of vaccinated participants in each cohort. With 114 clearance events among high-grade disease patients (n=126; vaccinated proportion ≈0.55) and 213 among low-grade patients (n=277; vaccinated proportion ≈0.47), results indicate that the study was only powered to detect relatively large effects (minimum detectable HR ≈0.61 for high-grade disease and ≈0.69 for low-grade disease with 80% power). When evaluating the adjusted vaccination effect size reported in the Cox model (HR ≈0.74), the achieved post-hoc power was approximately 23% in the high-grade disease group and 58% in the low-grade disease group, reinforcing suboptimal ability to detect smaller but potentially clinically relevant vaccine benefits in this context. Thus, the present study does not allow us to exclude potential positive effects of 9vHPV beyond primary prevention, requiring a larger sample and long-term prospective design to diligently analyse this matter.

## Conclusion

CC is a matter of public health concern, affecting many countries worldwide, including Portugal. Alongside CC screening, HPV vaccination is widely regarded as the most successful and cost-effective strategy for prevention of HPV infection and associated cancers. The importance of HPV vaccination among women with pre-existing infection is highlighted throughout currently available literature, but the rationale behind its efficacy in this setting remains unclear. The present study suggests limited benefit of 9vHPV as adjuvant measure in patients with active infection, emphasizing the importance of shared and informed decision-making when offering HPV vaccination to these women. Further research is currently being performed to better clarify the effectiveness of 9vHPV in reducing HPV-related disease burden among infected women, being crucial to determine the relevance of implementing HPV vaccination as part of secondary prevention strategies.

## Data Availability

The research data are described in the article presented.
